# Evolutions in Combined Heart-Kidney Transplant

**DOI:** 10.1007/s11897-024-00646-0

**Published:** 2024-01-17

**Authors:** Rashmi Jain, Michelle M. Kittleson

**Affiliations:** Department of Cardiology, Cedars-Sinai Medical Center, Smidt Heart Institute, 2nd floor, 8670 Wilshire Boulevard, Los Angeles, CA 90211 USA

**Keywords:** Heart failure, Kidney failure, Heart transplantation, Kidney transplantation

## Abstract

**Purpose of Review:**

This review describes management practices, outcomes, and allocation policies in candidates for simultaneous heart-kidney transplantation (SHKT).

**Recent Findings:**

In patients with heart failure and concomitant kidney disease, SHKT confers a survival advantage over heart transplantation (HT) alone in patients with dialysis dependence or an estimated glomerular filtration rate (eGFR) < 40 mL/min/1.73 m^2^. However, when compared to kidney transplantation (KT) alone, SHKT is associated with worse patient and kidney allograft survival. In September 2023, the United Network of Organ Sharing adopted a new organ allocation policy, with strict eligibility criteria for SHKT and a safety net for patients requiring KT after HT alone.

**Summary:**

While the impact of the policy change on SHKT outcomes remains to be seen, strategies to prevent and slow development of kidney disease in patients with heart failure and to prevent kidney dysfunction after HT and SHKT are necessary.

## Introduction

**Fig. 1:**
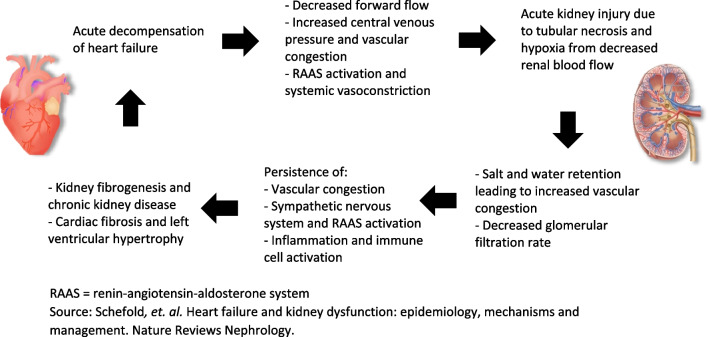
Bi-directional mechanisms leading to concomitant heart and kidney failure. RAAS = renin–angiotensin–aldosterone system. Source: Schefold*,* et al. Heart failure and kidney dysfunction: epidemiology, mechanisms and management. Nature Reviews Nephrology

**Fig. 2:**
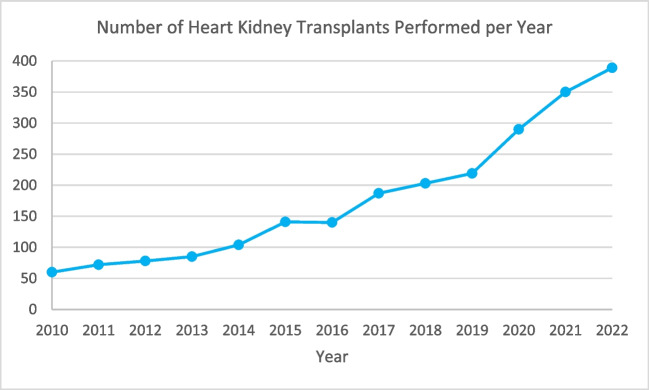
Number of heart-kidney transplants performed in the USA since 2010. Generated from data made available by Organ Procurement & Transplantation Network. https://optn.transplant.hrsa.gov/data/view-data-reports/national-data/

Heart failure and kidney disease commonly occur together due to multiple bi-directional mechanisms by which dysfunction in each leads to acute and/or chronic worsening of disease in the other (Fig. 1) [1]. In fact, approximately 40–50% of patients with heart failure with either reduced or preserved left ventricular ejection fraction (LVEF) carry a concomitant diagnosis of chronic kidney disease (CKD), as defined by estimated glomerular filtration rate (eGFR) of < 60 mL/min/1.73 m^2^. Any reduction in eGFR is strongly associated with increased mortality in heart failure patients [1, 2]. As such, the number of simultaneous heart-kidney transplants (SHKT) performed in the USA has grown approximately sixfold since 2010 (Fig. 2) [3].

While SHKT improves survival in patients with heart failure and kidney dysfunction compared to heart transplantation (HT) alone, it is by no means a panacea [4, 5, 6•, 7••]. Post-SHKT, a considerable proportion of patients experience severe acute kidney injury (AKI) requiring dialysis, and/or CKD over the long term, leading to increased post-transplant morbidity and mortality, and SHKT may reduce the lifespan of a kidney allograft [6•, 7••, 8, 9]. In this review, we discuss current and emerging strategies for management of SHKT candidates and patients, and examine the new SHKT organ allocation policy driven by ongoing ethical questions regarding organ allocation for SHKT.

## Outcomes After Heart-Kidney Transplantation

### Heart Transplant Outcomes in SHKT

The purpose of the pre-transplant kidney evaluation is to attempt to differentiate patients whose kidney disease may improve after HT alone from those whose disease will not improve and will benefit from SHKT [7••]. Observations from the United Network of Organ Sharing (UNOS) registry provide insight to guide this decision-making process. In one analysis of the UNOS database, in patients transplanted between 2003 and 2020 with reduced eGFR but not dialysis-dependence, recipients of SHKT versus HT alone had improved 5-year survival if eGFR was between 30 and 35 mL/min/1.73 m^2^. However, no survival advantage of SHKT vs HT was conferred in those with eGFR 35–45 mL/min/1.73 mL^2^ or higher. Of note, 1-year survival was similar between SHKT and HT alone regardless of eGFR [5]. Similar findings were observed in another UNOS registry analysis from 2005 to 2018, where those patients on dialysis or with an eGFR of 40 mL/min/1.73 mL^2^ or less had improved 5-year survival with SHKT vs HT alone [6•]. The benefit of SHKT versus HT alone in those with severe kidney dysfunction but not dialysis dependence was confirmed in a single-center analysis of 100 patients, where non-dialysis-dependent patients had comparable 15-year survival to those on dialysis prior to SHKT [4].

While these findings indicate the benefit of SHKT over HT alone in patients with severe kidney dysfunction, the results should be interpreted with caution for several reasons. These include variations in kidney function assessment. Kidney function may be measured inconsistently, with a variety of different thresholds, and there may also be a degree of survival bias since only patients stable enough for kidney transplant after HT were included in the SHKT groups [7••, 10].

Despite these pitfalls of observational analyses, the survival benefit observed with SHKT compared to HT alone in heart failure patients with dialysis dependence or reduced eGFR also extends to higher risk populations. In patients with obesity and reduced eGFR < 45 mL/min/1.73 m^2^ but not dialysis dependence, 5- and 10-year survival was higher with SHKT compared to HT alone, and SHKT also conferred a lower risk of rejection within the first year [11]. Similarly, in patients with eGFR < 45 mL/min/1.73 m^2^ undergoing heart retransplant evaluation, SHKT was associated with significantly improved survival at 1, 3, and 5 years, compared to repeat HT alone [12]. The survival advantage of SHKT is observed regardless of age (older or younger than 60 years of age) or sensitization (pre-transplant panel reactive antibodies of < 10%, 10–50%, or > 50%), indicating that even higher-risk HT candidates have improved outcomes with SHKT [4].

Of note, in addition to survival benefit, recipients of SHKT have a decreased risk of rejection and coronary allograft vasculopathy [7••, 8, 13–15]. There are multiple possible factors that may explain this observation, including the fact that kidney tissue has significantly higher HLA antigen levels than heart tissue [16]; additionally, in animal models, donor kidneys may possess cells that migrate to the host thymus and induce tolerance to donor antigens [14, 17, 18].

### Kidney Transplant Outcomes in SHKT

Despite favorable outcomes with SHKT compared to HT alone in patients with reduced eGFR, there remains a significant risk of post-SHKT kidney dysfunction. After SHKT, risks of severe AKI requiring dialysis are higher than after HT alone [7••]. In a single-center analysis of 35 patients, 37% of patients experienced delayed graft function, defined as need for dialysis within the first 7 days post-transplant, after SHKT, while another analysis found that 26% of patients required dialysis within 30 days after SHKT [9, 19]. In contrast, after HT alone, rates of early post-transplant dialysis range from approximately 7 to 22% [7••].

Risk factors for delayed kidney graft function after SHKT include higher donor age and increased pre-transplant body mass index, as well as elevated pre-transplant serum creatinine level [9, 20]. Risk factors for dialysis requirement at 1 year post-SHKT include elevated pre-transplant serum creatinine and redo transplantation [20].

Rates of kidney allograft survival at 1 year post-SHKT are also significantly lower when compared to kidney transplant alone, largely driven by worse 1-year patient survival, which is approximately 96% after kidney transplant alone, but ranges from 62 to 84% after SHKT [9, 17, 21]. The need for post-operative extra-corporeal membrane oxygenation, hemodynamic instability requiring intensive care unit management, and dialysis are predictors of post-operative mortality after SHKT [21, 22]. One analysis compared rate of kidney allograft failure in patients who received SHKT with that in the recipients of the donors’ contralateral kidneys, and found that after SHKT, 1-year rate of allograft failure was 5.8%, compared to 2.8% in the recipients of the contralateral kidneys; a similar pattern was observed for 1-year and 5-year rates of kidney allograft loss [6•]. The higher risk of kidney dysfunction after SHKT is concerning, as the presence of post-transplant AKI and/or CKD is associated with decreased short-term and long-term survival and increased risk of rejection in patients after HT alone [7••].

## Current UNOS SHKT Allocation Policy

In 2019, the American Society for Transplantation organized a consensus conference to establish national standards for eligibility for SHKT. As reflective of findings from the aforementioned analyses, consensus recommendations suggested that patients with eGFR < 30 mL/min/1.73 m^2^ may be considered for SHKT; those with eGFR > 45 mL/min/m^2^ likely may not be appropriate for SHKT, and those with eGFR 30–44 mL/min/1.73 m^2^ should be evaluated on an individual basis [23]. These thresholds were guidelines, not UNOS-enforced policies, and individual centers made the decision whether to list a HT candidate for a simultaneous kidney transplant. In this setting, the organ procurement organization would offer a kidney along with the heart to candidates listed at inpatient status and within 500 nautical miles of the donor hospital.

Despite these guidelines and evidence demonstrating the lack of benefit from SHKT in patients with eGFR > 40 mL/min/1.73 m^2^, there was significant center-specific variation in the listing of SHKT candidates, with a considerable proportion of SHKT recipients with a pre-transplant eGFR > 40 mL/min/1.73 m^2^ [6•, 10]. The fact that these SHKT candidates would have priority for kidney transplantation over those awaiting a kidney-only transplant appeared to violate the goal of maximizing utility of a limited resource, and thus a better policy was sought.

On September 28, 2023, UNOS implemented new explicit criteria for SHKT allocation with a safety net policy to promote access to transplantation for patients who receive a HT and later need a kidney transplant [24••]. The purpose of these new criteria was to achieve the best use of scarce donor organs by improving equity in transplant opportunities for multi-organ and single-organ candidates. In the new policy, the kidney is only offered along with the heart to HT candidates who meet a specific level of kidney dysfunction. For those who meet the criteria, the kidney is now offered to all HT candidates, not just those with inpatient listing status. The new medical eligibility for simultaneous heart-kidney allocation includes either evidence of CKD or sustained AKI (Table 1). To meet qualifications for CKD, defined as eGFR < 60 mL/min/1.73 m^2^ for greater than 90 consecutive days, there must be documentation of need for dialysis or creatinine clearance less than 30 mL/min; for sustained AKI, need for dialysis at least once every 7 days or creatinine clearance < 25 mL/min at least once every 7 days must be documented for a period of 6 weeks.

**Table 1:** Simultaneous heart-kidney transplant and safety net eligibility criteria

Criterion	Definition	Allocation policy
CKD	eGFR < 60 mL/min for > 90 consecutive days with one of: - ESRD with regular dialysis - CrCl or eGFR < 30 mL/min on or after date of kidney waiting list registration	A heart and kidney available from the same donor should be offered to a candidate who meets one of these criteria, is registered within 500 nautical miles of the donor hospital, and is listed adult heart status 1–5, before the kidney is offered to a kidney-alone candidate
Sustained AKI	For a period of 6 weeks, either one or a combination of: - Dialysis requirement at least once every 7 days - CrCl or eGFR < 25 mL/min at least once every 7 days	
Prior heart recipient safety net	Meets both of: 1. Registered on kidney waiting list within 1 year of HT date 2. Between 60 and 365 days after HT, is either on dialysis, or has CrCl or eGFR ≤ 20 mL/min	A candidate who meets this criterion receives priority on the kidney-alone waiting list

*AKI*, acute kidney injury; *CKD*, chronic kidney disease; *CrCl*, creatinine clearance; *eGFR*, estimated glomerular filtration rate; *ESRD*, end-stage renal disease; *HT*, heart transplant; *SHKT*, simultaneous heart kidney transplant

Source: OPTN Notice of Policy Changes to Establish Eligibility Criteria and Safety Net for Heart-Kidney and Lung-Kidney Allocation, September 28, 2023

For those who no longer meet the criteria for SHKT, there is a safety net for them to receive some priority in kidney allocation if they meet kidney transplantation criteria within the first year after HT. The safety net policy applies to those patients who received a HT alone and then develop kidney dysfunction after HT. HT recipients qualify for priority in kidney transplant allocation under the safety net policy if they have an estimated eGFR 20 mL/min/1.73 m^2^ or less or are on dialysis anytime between 60 and 365 days after HT.

Experience with safety net policies is available based on that established for liver-kidney transplant recipients since 2017. Since 2017, there has been a 16% decrease in simultaneous liver-kidney transplants with an increase in kidney after liver transplants [25], suggesting better use of donor organs. A safety net approach also theoretically allows for living kidney donation which not only has superior outcomes than a deceased kidney donor but also increases the overall donor pool. However, the living kidney donation rate is low, with only 10 living kidney transplants after liver transplantation between August 30, 2017 and December 31, 2019 [26], making the widespread feasibility of this option unclear.

With implementation of the new restrictive policy for SHKT allocation in September 2023, it remains to be seen if outcomes of HT recipients with severe kidney dysfunction not meeting SHKT eligibility remain favorable, and the frequency of use and outcomes of kidney transplantation under the safety net policy.

## Optimizing SHKT Outcomes

### Pre-Transplant Care and Management

An important strategy to optimize outcomes of patients with advanced heart failure and concomitant kidney disease involves guideline-directed medical therapy to preserve or improve kidney function [27–30]. In heart failure with reduced ejection fraction (HFrEF), the pillars of GDMT include beta-blockers, angiotensin-converting enzyme inhibitors (ACEi)/angiotensin receptor blockers (ARB)/angiotensin receptor and neprilysin inhibitors (ARNI), mineralocorticoid receptor antagonists (MRA), and sodium-glucose cotransporter-2 (SGLT2) inhibitors [31]. The majority of landmark randomized clinical trials supporting use of these medications in HFrEF excluded patients with advanced CKD, but evidence of their benefits in patients with CKD, and even in some cases dialysis dependence, continues to grow.

Beta-blockers do not impact GFR and tend to be well-tolerated; in patients with HFrEF on dialysis, one trial demonstrated a mortality benefit with carvedilol [32]. Both ACEi and ARBs improve outcomes in patients with heart failure and CKD in post hoc analyses of landmark trials [30]. In patients who also have diabetes in addition to HF and CKD, use of ACEi/ARB slows progression of GFR decline. More recently incorporated into practice, ARNIs decrease the risk of a sustained 50% reduction in GFR or development of end-stage kidney disease and slow the annual decrease in GFR in heart failure with reduced or preserved LVEF. SGLT2 inhibitors also slow annual decline in GFR; they also have a significantly lower rate of the composite renal endpoint of 50% sustained decline in GFR, end-stage kidney disease, or kidney-related death, in a meta-analysis of landmark trials for dapagliflozin and empagliflozin [30]. However, many patients being considered for SHKT are either unable to tolerate optimal GDMT due to hypotension or electrolyte abnormalities, or have continued to experience progression of disease despite these therapies.

In patients with more advanced disease, temporary mechanical circulatory support (tMCS) is increasingly employed to bridge patients to transplant. In 2018, UNOS modified its heart allocation policy to allow for prioritization of sicker patients, such as those on tMCS [33]. Since that change, the numbers of patients who received SHKT via tMCS, including intra-aortic balloon pump (IABP), catheter-mounted continuous micro-axial flow pump (such as the Impella pump, Abiomed, Danvers, Massachusetts), and veno-arterial extra-corporeal membrane oxygenation (VA-ECMO) has grown significantly [34–37]. The impact of use of pre-transplant tMCS on post-SHKT outcomes has remained equivocal. One analysis found that use of IABP, Impella, and VA-ECMO did not affect 1-year SHKT survival, with similar survival between patients who were bridged with tMCS and those who were not [34]; similarly, another examination of the risk factors for post-SHKT acute kidney injury and need for dialysis at 1 year post-transplant found no relationship between use of tMCS and these outcomes [20].

However, multiple other analyses demonstrated that bridge to transplant with VA-ECMO was associated with worse survival after SHKT, as well as increased risk of delayed kidney graft function and kidney allograft failure [36, 37]. When SHKT recipients on pre-transplant tMCS were compared with kidney transplant-only recipients of the contralateral kidneys of the same donors, bridging with MCS was associated with a twofold increased risk of kidney allograft failure [6•]. Although patients with advanced CKD and ESRD are not typically candidates for durable MCS such as left ventricular assist devices (LVAD), one analysis demonstrated that patients bridged to SHKT from durable LVAD had worse 1-year and 5-year survival, and were more likely to require post-SHKT dialysis, than those who were bridged to HT alone [38].

The impact of the UNOS heart allocation policy change on overall outcomes after SHKT has been similarly mixed. Some studies have demonstrated that waitlist outcomes for SHKT candidates have improved with the advent of the change, with decreased death and de-listing, increased rates of transplant compared to the old system, and faster time to transplant for patients listed at higher urgency status [34, 37]; however, not all studies came to the same conclusion [35]. When examining changes in mortality with the UNOS policy change, some studies showed worse survival and increased kidney and heart graft failure under the new system, while others demonstrated no difference in survival after the change [34–37]. Other factors aside from tMCS that may explain differences in outcomes after the policy change include overall increase in total organ ischemic time with the new system based on broader geographic sharing, and a higher proportion of patients that were not on dialysis at the time of SHKT [34, 36, 37].

### Surgical Considerations

The major consideration in surgical planning for SHKT is the timing of the kidney transplant. It can either be performed as a single operation, with the kidney implanted immediately after HT, or in a staged fashion, with kidney implantation performed after a delay. The preferred approach is performing the operation in a staged manner, with a short delay between the two organ implants, to allow for hemodynamic stabilization before kidney implantation [7••, 39]. Activation of the inflammatory cascade during cardiopulmonary bypass, use of vasoconstrictors, and hemodynamic instability in the immediate post-HT period, may all negatively impact kidney allograft function [39].

Although multicenter, prospective, or randomized studies directly comparing the two methods are lacking, single-center analyses and small case series have demonstrated comparable long-term survival and rejection outcomes with the staged method to HT alone [39], as well as acceptable immediate and long-term kidney allograft function [40]. In these reports, kidney implantation was delayed as long as 48 h, with total kidney cold ischemic time of up to 64 h, without significant negative impact on kidney allograft function [40]. The additional benefit of delaying kidney implantation is that in patients who are too hemodynamically unstable after HT with a high risk of morbidity and mortality, the kidney allograft could potentially be implanted into an alternative recipient [40].

### Immunosuppression

Considerations for immunosuppression in SHKT patients include whether to utilize an induction regimen and how to optimize the maintenance regimen to avoid excessive nephrotoxicity. Induction therapy regimens and practices vary greatly by institution, but it is generally preferred in order to allow for delayed initiation of the nephrotoxic calcineurin inhibitors (CNI) [7••]. The most common induction strategies are lymphocyte-depleting antibodies, usually polyclonal rabbit anti-thymocyte globulin (ATG), and interleukin-2 receptor antibodies (IL2RA) [41]. In one analysis of the UNOS registry comparing SHKT recipients who received no induction, induction with ATG, and induction with IL2RA, there was no difference in kidney allograft function or acute rejection of the heart or kidney within the first year of transplant. However, SHKT recipients who received ATG induction had improved post-transplant survival, and on multivariable analysis, the difference was significant in patients who were previously sensitized, with a PRA > 10% [41].

Maintenance immunosuppression typically consists of a CNI, antimetabolite agent, and a corticosteroid. Tacrolimus is the preferred calcineurin inhibitor compared to cyclosporine, due to less rejection, nephrotoxicity, hyperlipidemia, hypertension, and diabetes [42–44]; for the antimetabolite agent, mycophenolate mofetil is preferred over azathioprine due to reduced treated rejection and mortality at 1 year [45]. Proliferation signal inhibitors (also known as mammalian target of rapamycin (mTOR) inhibitors) also reduce acute rejection and prevent development of cardiac allograft vasculopathy in heart transplant recipients [46–48], but are not initiated immediately after HT due to potentiation of the nephrotoxic effects of calcineurin inhibitors and poor wound healing [49]. However, when proliferation signal inhibitors replace calcineurin inhibitors after 3–6 months post-transplant, transplant recipients experience slower progression of kidney dysfunction, with improved kidney function at 1 year. This benefit must be balanced with the increased risk of biopsy-proven rejection observed in patients maintained on CNI-free regimens [7••, 50, 51].

One approach to mitigate the increased risk of rejection from a CNI-free regimen might be photopheresis. In a retrospective analysis of HT recipients who received photopheresis for primary prevention of acute rejection after HT, 88% remained free from rejection over the subsequent 26 months, and treatment efficacy was not compromised by reduction in CNI exposure [52]. However, the use of photopheresis in primary prevention has not entered standard practice due to the intense resource utilization of this approach.

A newer advance to minimize CNI toxicity is belatacept, a selective T-cell co-stimulation blocker [7••]. In kidney transplant recipients, this recombinant immunoglobulin fusion protein was associated with better patient and graft survival, higher eGFR, and decreased de novo donor specific antibody formation when compared with CNI-based immunosuppression regimens [53–55]. However, belatacept is not currently approved for use in HT recipients by the US Food and Drug Administration, given insufficient data to support this approach in these patients [7••, 56]. In the future, it may be an effective strategy to decrease nephrotoxicity from CNIs in SHKT recipients. Continued study for identifying strategies to prevent rejection and promote renal protection is warranted.

## Conclusion

As rates of SHKT continue to grow, there is evidence to suggest that in patients with concomitant heart failure and chronic kidney disease, SHKT may lead to superior outcomes when compared with HT alone. However, to address potential inequities in the allocation of kidney transplantation, UNOS implemented strict criteria for SHKT listing in September 2023 with a safety net policy to prioritize kidney transplantation for those HT recipients with severe kidney dysfunction early after HT. While optimization of pre-transplant, peri-operative, and post-transplant management will maximize outcomes in recipients of these dual-organ transplants, there remains a need for strategies to prevent or slow progression of kidney disease in heart failure patients, and to limit AKI and CKD post-transplantation. Finally, assessment of post-SHKT outcomes with the new safety net policy will facilitate continued improvement of organ allocation policies.
